# Development of user‐selectable diverse sets of cultivated and wild soybean germplasm for genetic and breeding applications

**DOI:** 10.1002/tpg2.70216

**Published:** 2026-03-09

**Authors:** Qijian Song, Susan Araya, Chuck Quigley, Patrick Elia

**Affiliations:** ^1^ USDA‐ARS, Soybean Genomics and Improvement Lab, Beltsville Agriculture Research Center Beltsville Maryland USA

## Abstract

After decades of intensive breeding, modern US soybean [*Glycine max* (L.) Merr.] varieties have achieved significant improvements in yield, quality, and stress tolerance, but these gains have come at the cost of severely reduced genetic diversity. To reduce vulnerability and promote efficient use of germplasm, diverse sets (DS) of varying sample sizes were defined for the entire USDAARS Soybean Germplasm Collection and 13 maturity groups using the SoySNP50K single‐nucleotide polymorphism (SNP) profile. The average retained genetic diversity of the 50K SNPs was then compared between 10 DS and 10 random sets (RSs) at different sizes. DS consistently outperformed random sampling: in cultivated soybean, DS captured 94.9%–98.4% of SNP diversity compared with 73.1%–93.9% for RS; in wild soybean, DS captured 92.8%–97.9% compared with 83.4%–97.7% for RS. The performance of DS was further validated using whole‐genome sequences from 1511 accessions, demonstrating that DS could retain the diversity predicted by the SNP subset across 1308 cultivated and 203 wild soybean genomes of different sample sizes. DS was also effective in capturing genetic diversity across different traits. To allow users to select DS, a “Soy‐DS Selector” approach was proposed, and a table containing germplasm clusters across the USDA collection and different maturity groups was created. This resource enables researchers to tailor combinations based on maturity groups, accession and sample size preferences, and seed availability. The study provides both methodology and resources that can streamline germplasm evaluation, maximize resource utilization, and enhance future genetic improvement in soybean. Several DS have already been used by US soybean breeders in their programs.

## Core Ideas

- Methodology and resources were provided that can streamline germplasm evaluation, utilization, and enhance soybean improvement.
- Diverse sets of soybean germplasm were developed for the entire USDAARS Soybean Germplasm Collection and for different maturity groups.
- A Soy‐DS Selector table was created that allows users to easily build their own custom diverse sets based on maturity group, sample size, preferred germplasm, and so forth.
- Several soybean diverse sets have already been used by US soybean breeders for genetic or breeding research.

## Plain Language Summary

US soybean breeding has long improved yield, quality, and resistance but sharply reduced genetic diversity—over 95% of modern lines come from only a few ancestors. To address this loss, researchers analyzed ∼20,000 cultivated and wild soybean accessions using ∼50,000 genetic markers to measure how genetically different each accession is from others. They built genetic diversity sets that capture the broadest possible variation and found that 300–400 well‐chosen accessions can represent most diversity in the entire collection. To support breeders, the team created a “Soy‐DS Selector” table, a simple tool that lets users generate customized germplasm diverse sets based on location, preferred materials, or seed availability. This approach gives breeders and researchers an efficient way to preserve, explore, and use soybean diversity for soybean improvement.

**Abbreviations**
DS: diverse sets
GWAS: genome‐wide association study
MG: maturity group
RS: random set
SNP: single‐nucleotide polymorphism

## 1 INTRODUCTION

During centuries of cultivation and selection, especially the past six or seven decades of intense selection imposed by breeding programs, soybean [*Glycine max* (L.) Merr.] seed yield, quality, and resistance to biotic and abiotic stresses have dramatically improved. However, the genetic diversity of elite soybean cultivars has declined consequently. Generally, all modern US soybean commercial cultivars can be traced back to a limited number of ancestors. A parentage study showed that 28 ancestors and seven first progenies contributed 95% of the pedigree found in 258 modern US cultivars released from 1947 to 1988 (Gizlice et al., 1994). Of the 494 soybean cultivars registered in North America through the US Plant Variety Protection Office and in scientific journals from 1999 to 2008, 23% have a genetic contribution of at least 25% from cultivar A3127 (Mikel et al., 2010). A molecular study revealed that modern cultivars have retained 72% of the sequence diversity present in the Asian landraces from which they derive but have lost 79% of the rare alleles found in the Asian landraces (Hyten et al., 2006). The most significant bottleneck was the domestication process, during which cultivated varieties retained only 66% of the nucleotide diversity of wild soybean (*Glycine soja*) and lost most of the rare alleles present in wild soybeans (Hyten et al., 2006). From wild species to landraces and then to improved varieties, genetic diversity decreased in terms of haplotype number, haplotype block size, and quantity (Song et al., 2015). Limited genetic diversity in soybean will result in its vulnerability to disease, insects, and other stresses and jeopardizes the potential for sustained genetic improvement. Therefore, researchers began to introduce germplasm resources of wild soybeans, exotic soybeans, and local soybean varieties into breeding programs, which led to the development of new germplasm resources (Bagherzadi et al., 2020; Fallen et al., 2024; Stewart‐Brown et al., 2020; Viana et al., 2022). USDA‐ARS has collected and preserved more than 1100 wild soybean accessions from China, Japan, Korea, and Russia, and >18,000 cultivated soybean accessions from China, Japan, Korea, and 84 other countries (Song et al., 2015). Within this collection, a total of 30% *G. soja* and 23% *G. max* have greater than 99.9% similarity, respectively (Song et al., 2015). Among these, 8% of the *G. soja* and 9% of the *G. max* accessions were 100% identical to at least one other accession based on the analysis of the SoySNP50K dataset. The high rate of duplication or similarity of genotypes in the USDA collection is one of the reasons for developing soybean diverse sets (DS). The DS of accessions will represent the entire/target accessions with minimum repetitiveness and maximum genetic diversity, increase the efficiency of germplasm characterization, and facilitate the utilization of genetic resources in soybean breeding programs.

Soybean seed yield and yield stability are threatened by biotic stresses such as pests and diseases, and abiotic stresses such as drought, waterlogging, high temperature, salinity, and ozone. Developing varieties that are resistant or tolerant to abiotic and biotic stresses requires germplasm containing resistance genes or elements. The identification of the desired germplasm often requires screening of a large number of accessions under stress conditions. Due to limitations in screening capacity and resources, breeders can only evaluate a certain number of accessions in the field or greenhouse. Therefore, identifying diverse germplasm sets with different sample sizes will facilitate evaluation and provide breeders with options to make decisions based on their own capabilities and needs and help them to improve evaluation efficiency.

Genome‐wide association studies (GWASs) are a common approach to identify genes/quantitative trait loci (QTLs) controlling different traits (Bandillo et al., 2015; Hwang et al., 2014; Vuong et al., 2015; J. Zhang et al., 2016). GWAS leverage historical recombination events captured in natural populations to achieve fine‐scale mapping resolution, rather than relying solely on recent recombination from controlled crosses. This approach facilitates QTL/gene mining by using natural germplasm populations as material rather than recombinant inbred line populations that require crosses and years of development. Utilizing DS, rather than random germplasm, will improve the statistical power of GWAS by reducing redundant germplasm and increasing the range of phenotypic variations in the population.

As a short‐day and temperate plant, soybean is sensitive to photothermal conditions during flower initiation and development (Cober et al., 1996; Major et al., 1975). The responses of soybean varieties to photothermal conditions determine the zone of their adaptation, and can affect other traits, such as plant height, yield, seed quality, and so forth (Cooper, 2003). While some flexibility exists, the range of adaptation is quite narrow—typically only one or two maturity groups (MGs) are suitable for a specific region, which is usually the case for most breeding seed yield trials. Thirteen major growing zones or MGs have been recognized for soybean, ranging from MG 000 to MG X. Soybeans in MG 000 are adapted for growth in upper North American regions, soybeans with an MG X are adapted for growth in tropical regions. When developing soybean DS, germplasm selection must also consider the MG of the geographic region, so the soybean DS can grow, develop, and mature normally under natural field conditions.

Practical challenges often arise when using collections: some accessions lack sufficient seed for planting, are unavailable from active gene banks, or exhibit poor germination and require replacement. Researchers may also wish to include preferred accessions. These scenarios must be considered when developing the DS. Prior to the formation of the DS, a procedure of stratifying the sample must be established based on either ecogeographic origin or phenotypic or genotypic information. The standard procedure of stratification is to maximize the diversity among clusters while minimizing the diversity within clusters. Once the clusters are formed, an allocation method is used to determine the number of accessions to be drawn from each cluster (Kim et al., 2007; Thachuk et al., 2009). A diverse collection generally is required to contain 5%–10% of the germplasm collection (Brown, 1989; Diwan et al., 1995; Ortiz et al., 1998; Qiu et al., 2003; Yan et al., 2007; Zhao et al., 2005) and ideally conserves at least 70% of the alleles in the whole collection (Brown, 1989). Core collection sets, a type of DS, have been developed in many crops, such as rice (Chung et al., 2009; Damirgi et al., 1967; de Oliveira Borba et al., 2009; Lou et al., 2011), wheat (Balfourier et al., 2007; Kobiljski et al., 2002; Martynov et al., 2003), barley (Lasa et al., 2001; Liu et al., 2002; Ottosson et al., 2002), maize (Coimbra et al., 2009; Flint‐Garcia et al. 2005; Malvar et al., 2004), and other species. In soybean, core collections of 3000 accessions for *G. max* (Qiu et al., 2003) and 652 accessions for *G. soja* (Zhao et al., 2005) were developed in China, respectively. Oliveira et al. (2010) developed a *G. max* USDA soybean core collection with 1685 accessions using a multivariate proportional selection strategy based on 29 morphological and quality traits, including flower color, growth habit, origin, lodging score, maturity, seed shattering score, seed size, seed yield, protein content, oil content, and other seed compositions. While the core set may capture the diversity of the traits originally considered, it is unclear whether it also represents the diversity of other important traits, such as stress tolerance and resistance to diseases and pests, which were not included when the core collection was established. Furthermore, the measurement of certain traits—particularly quantitative traits—can be influenced by environmental factors, which may affect the development and representativeness of the core sets. A core collection of *G. soja* with 192 accessions was also reported in Japan based on a limited number of 20 SSR markers (Kuroda et al., 2009). However, these core collections were constructed based on only a very limited number of agronomic traits or molecular markers, as well as information on the geographic origin of the germplasm. The USDA Soybean Germplasm Collection contains approximately 20,000 wild and cultivated soybean accessions that were genotyped by the SoySNP50K BeadChip assay (Song et al., 2015; Song et al., 2012). This dataset provides a more complete resource for correctly defining diverse germplasm collections. Our goal was to assemble diverse soybean accessions with the sample sizes that are most used or manageable by soybean researchers, and allow them to select their own DS (termed “Soy‐DS Selector”) based on their preferred accessions, seed availability, and capacity to manage or evaluate the germplasm, leading to more efficient and economical utilization of soybean germplasm resources.

**Core Ideas**

- Methodology and resources were provided that can streamline germplasm evaluation, utilization, and enhance soybean improvement.
- Diverse sets of soybean germplasm were developed for the entire USDAARS Soybean Germplasm Collection and for different maturity groups.
- A Soy‐DS Selector table was created that allows users to easily build their own custom diverse sets based on maturity group, sample size, preferred germplasm, and so forth.
- Several soybean diverse sets have already been used by US soybean breeders for genetic or breeding research.

## 2 MATERIALS AND METHODS

### 2.1 Genotyping USDA Soybean Germplasm Collection

The USDA Soybean Germplasm Collection consists of approximately 20,000 accessions including 1168 *G. soja* from China, Korea, Japan, and Russia, and 18,484 *G. max* from China, Korea, Japan, the United States, and other countries. The germplasm was genotyped with the SoySNP50K BeadChip assay (Song et al., 2015; Song et al., 2012). The 42,509 single‐nucleotide polymorphisms (SNPs) in the SoySNP50K assay were selected from euchromatic and heterochromatic regions of the 20 soybean chromosomes and had physically even spacing between adjacent SNPs in the euchromatic and heterochromatic regions, where applicable, as described by Song et al. (2015). The SoySNP50 dataset was deposited at SoyBase (https://soybase.org/dlpages/#snp50k.) for public access.

### 2.2 Procedure for the selection of soybean DS

The selection procedures for DS were as follows: (A) Pairwise genetic distances were calculated among 18,484 *G. max* and 1168 *G. soja* soybean accessions, respectively, based on polymorphic SNPs generated by the SoySNP50K assay (Song et al., 2015). Pairwise genetic distance matrices were also calculated for accessions used to develop the DS of each MG. For each MG, distance calculations included accessions from that MG as well as accessions from an early MG and a late MG, for example, for MG 00, accessions from MG 000, MG 00, and MG 0 were included, for MG 0, accessions from MG 00, MG 0, and MG I were included, and so on, because accessions from immediately adjacent MGs can usually flower and mature normally. However, for MG 000 (the earliest MG), only accessions from MG 000 and MG 00 were included, and for MG X (the latest MG), only accessions from MG IX and MG X were included. (B) Accessions were clustered, with the number of clusters corresponding to the typical final number of accessions that users may want to use for various research purposes, that is, 25, 50, 100, 200, 300, 400, 500, 600, 700, 800, 900, 1000, and equal to 10% of the number of accessions in the entire *G. max* collection and 10% of the number of accessions in each MG. No DS selection was performed for each MG of *G. soja* due to the low number of accessions in the MGs. (C). Within each cluster, one accession was randomly selected or preferentially chosen (i.e., any accession the user wishes to include) to form a diverse set. Pairwise genetic distance calculation and partitioning individual accessions into clusters were performed using PLINK with the *k*‐means method (Purcell et al., 2007). In general, PLINK calculated genetic distances between all individuals based on allele sharing, and iteratively assigned individuals to clusters based on their average genetic similarity to existing clusters. This process continued until the assignments were stable (converged), meaning that the assignments no longer changed significantly. The *k*‐means method is particularly useful in identifying related individuals or subpopulations in a dataset. The following procedures and PLINK commands were used to group the germplasm into *n* clusters:

Step 1: Create a PED file (e.g., input.ped) with genotypic data and a MAP file (e.g., snp_position.map) with SNP positions, following PLINK instructions.
Step 2: Convert PED (input.ped) and MAP (snp_position.map) files into binary format (BED, BIM, FAM) and output the file (e.g., out_bfile):
plink –ped input.ped –map snp_position.map –make‐bed –out out_bfile
Step 3: Generate the IBS (Identity‐by‐State) matrix (e.g., out_IBS_matrix) using out_bfile:
plink –bfile out_bfile –genome –out out_IBS_matrix
Step 4: Perform clustering into *n* clusters (e.g., 200) and output cluster file (e.g., cluster_file): plink –bfile out_bfile –read‐genome out_IBS_matrix.genome –cluster –K 200 –out cluster_file. The output will include a cluster file: cluster_file.cluster2.

### 2.3 Comparison of SNP number and percentage of polymorphic SNPs retained in the DS and random sets

To compare the number and percentage of polymorphic SNPs captured by the DS versus random selection without clustering, a total of 10 random sets (RSs) of random accessions without considering clustering and 10 DS of accessions with one accession selected randomly from each cluster were iteratively generated to calculate the number of SNPs retained and the percentage of SNPs retained from the 42,509 SNPs in the SoySNP50K dataset for each RS and diverse set, and the maximum number, the minimum number, and the standard deviation of the SNPs captured among the RS and DS. The comparison and analyses of DS and RS were performed for each predefined cluster sizes of 25, 50, 100, 300, 400, 500, 600, 700, 800, 900, 1000 and 1849 for *G. max* and 25, 50, 100, 116, 300, 400, 500, 600, 700, 800, 900 and 1000 for *G. soja*. The number of SNPs, the percentage of SNPs retained from the SNPs presented in germplasm of each MG, and the maximum number, the minimum number, and the standard deviation of the SNPs captured by the 10 DS and 10 RS were also calculated for each *G. max* MG at sample size of 25, 50, 100, 300, 400, 500, 600, 700, 800, 900, 1000, and 10% of the total accessions for the MG if applicable.

### 2.4 Comparison of genetic diversity retained in the soybean DS based on partial SNPs and all SNPs obtained from 1511 soybean whole‐genome sequences

To test the validity and reliability of the selection method, we analyzed a whole‐genome sequence dataset of 1511 soybean accessions (Liu et al., 2025; Zhang et al., 2022). Based on the whole‐genome sequences of these 1511 accessions, 6,230,008 and 13,311,173 SNPs with MAF greater than 0.01 were identified in 1308 *G. max* and 203 *G. soja* accessions, respectively. Among them, 42,509 SNPs were randomly selected from wild soybean, and 42,509 SNPs were independently selected from cultivated soybean, and 10 DS and 10 RS were constructed using these 42,509 SNPs, with sample sizes of 10, 25, 50, and 100, respectively. The number of SNPs and the percentage of SNPs retained were calculated based on the 42,509 SNPs. In order to observe the actual genetic diversity retained by these DS and RS in the whole genome and to verify whether the genetic diversity predicted by DS based on the SNP subset can be used as an estimate of the genetic diversity of the whole genome, the retained number of SNPs and the percentage of SNPs of the DS and RS were also analyzed based on 6,230,008 SNPs of *G. max* and 13,311,173 SNPs of *G. soja* in the DS and RS, respectively.

### 2.5 Comparison of morphological, agronomic, and seed composition traits among entire collection and DS

To examine whether DS captures trait diversity, we calculated means and ranges for quantitative traits and trait percentages for qualitative traits for DS of 1849 *G. max* accessions and compared these results with the results for all 18,484 *G. max* accessions. We also calculated these trait statistics for DS of 116 *G. soja* accessions and compared these results with those for all 1168 *G. soja* accessions. Morphological, agronomic, seed composition, and other traits of the USDAARS Soybean Germplasm Collection were downloaded from SoyBase and GRIN‐Global (https://legacy.soybase.org/grindata/results.php). These traits were recorded across different locations and/or years for various accessions. Since some germplasm materials were missing phenotypic data, the calculation of traits was based on the subset of accessions with available data.

### 2.6 Genetic distance analysis of 1685 USDA core soybean accessions selected by phenotypic traits

A USDA core collection of 1685 soybean accessions was selected by Oliveira et al. (2010). Pairwise genetic distances among these accessions were calculated using 42,509 SNPs from the SoySNP50K assay (Song et al., 2015; Song et al., 2012), with the formula:

$$d_{ij}=\frac{\sum g_{ij}}{N}$$

where $d_{ij}$ = genetic distance between the *i*th and *j*th accessions and $g_{ij}$ = count of SNP loci with different alleles; If an SNP allele is missing in either accession, that locus is excluded from the calculation. If one accession is heterozygous and the other homozygous, the difference counts as 0.5; if both are heterozygous, it counts as 1.N = total number of SNP loci without missing data in both accessions.

## 3 RESULTS

### 3.1 Redundancy of accessions in the USDAARS Soybean Germplasm Collection

Among the 18,484 *G. max* accessions, the number of accessions in each MG ranged from 106 in MG X to 4232 in MG IV. Among the 1168 *G. soja* germplasm materials, the three MGs MG 000, MG V, and MG VI each had more than 100 accessions, while the MGs MG VIII, MG IX, and MG X each had a small number of accessions (Table 1).

**TABLE 1:** Number of *G. max* and *G. soja* accessions in the USDAARS Soybean Germplasm Collection.

MG	Number of *G. max* accessions	Number of *G. soja* accessions	MG	Number of *G. max* accessions	Number of *G. soja* accessions
000	141	108	V	2517	356
00	513	49	VI	1512	166
0	1116	52	VII	928	79
I	1673	60	VIII	947	1
II	2061	95	IX	726	3
III	1974	50	X	106	4
IV	4232	84	Undetermined	37	61

Abbreviation: MG, maturity group.

*G. max* and *G. soja* accessions were grouped based on the genetic similarity across the 42,509 SNP markers; a total of 1743 and 133 groups with >99.9% identity were obtained in *G. max* and *G. soja*, respectively. Among these, four groups in *G. max* and one in *G. soja* contained more than 50 accessions (Table 2). A total of 95 *G. soja* and 1682 *G. max* accessions were 100% similar to at least one other accession.

**TABLE 2:** Frequency of groups with accessions having similarity >99.9% within the group.

Number of accessions per group	Number of groups in *G. max*	Number of groups in *G. soja*
≥50	4	1
≥40 and <50	1	0
≥30and <40	3	1
≥20 and <30	19	1
≥10 and <20	50	3
≥3 and <10	561	36
2	1105	91
Total	1743	133

### 3.2 Comparison of percentage of polymorphic SNPs captured in the DS and RS of *G. max* and *G. soja*

**FIGURE 1:**
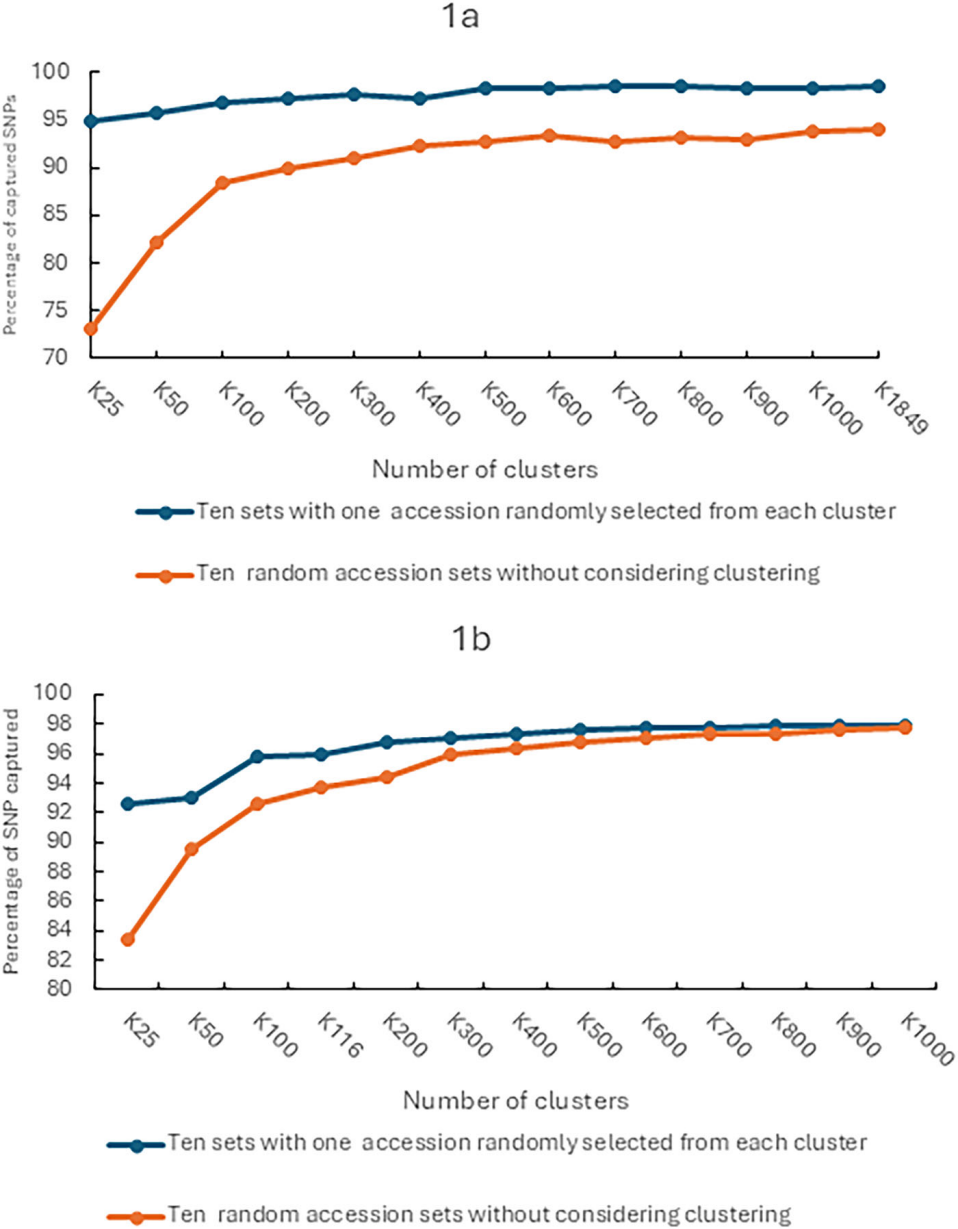
Comparison of the percentage of single‐nucleotide polymorphism (SNP) captured by completely random selection and by randomly selecting one accession from each cluster. (a) Percentage of *G. max* SNPs captured by 10 diverse sets constructed by randomly selecting one accession from each cluster (blue line), and 10 random sets constructed without considering cluster (orange line) at different sample sizes. (b) Percentage of *G. soja* SNPs captured by 10 diverse sets constructed by randomly selecting one accession from each cluster (blue line), and 10 random sets constructed without considering cluster (orange line) at different sample sizes.

We calculated the average number and percentage of polymorphic SNPs captured by 10 DS with and 10 RS without considering clusters at each sample size for the entire *G. max* and *G. soja*, respectively (Table S1, Figure 1). For *G. max*, the diversity captured in DS was 94.91%–98.40% when the sample size increased from 25 to 1000, while the diversity captured in RS was 73.10%–93.86% when the sample size increased from 25 to 1000. For the same sample size, the diversity captured by DS was significantly higher than that of RS. For *G. soja*, when the sample size increased from 25 to 1000, the diversity captured by the DS ranged from 92.75% to 97.86%, while the diversity captured by the RS method ranged from 83.36% to 97.74%, and the difference was significant when the sample size was smaller than 600. When the sample size was at 700 or above, the difference between RS and DS became insignificant (Figure 1), consistent with the observation by Song et al. (2015) that approximately 30% of the *G. soja* accessions had a similarity greater than 99.9%.

Within each MG, as the sample size increased, higher diversity was captured by both DS and RS. The diversity captured by all DS was significantly higher than that by RS at 1% or 5% significant probability levels except for MG VIII at a sample size of 1000 (Table 3, Table S2).

**TABLE 3:** Diversity captured by random sets and diverse sets with different sample sizes in each *G. max* maturity group.

Target maturity groups	*G. max* maturity groups whose accessions were included for selection	Percentage of diversity captured by diverse sets with sample sizes 25–1000	Percentage of diversity captured by random sets with sample sizes 25–1000	Significance of difference of diversity captured between diverse sets and random sets at different sample sizes
MG 00	MG 000, MG 00, and MG 0	90.42–96.48	81.17–94.95	All significant at 1% or 5% probability levels
MG 0	MG 00, MG 0, and MG I	91.00–95.92	82.94–95.00	All significant at 1% probability levels
MG I	MG 0, MG I, and MG II_	92.68–96.86	82.79–94.74	All significant at 1% probability levels
MG II	MG I, MG II, and MG III	92.20–96.44	83.40–94.85	All significant at 1% probability levels
MG III	MG II, MG III, and MG IV	91.71–96.61	84.29–94.89	All significant at 1% probability levels
MG IV	MG III, MG IV, and MG V	91.45–96.79	83.85–94.34	All significant at 1% probability levels
MG V	MG IV, MG V, and MG VI	91.73–96.78	82.98–93.82	All significant at 1% probability levels
MG VI	MG V, MG VI, and MG VII	89.92–95.19	80.37–94.14	All significant at 1% probability levels
MG VII	MG VI, MG VII, and MG VIII	89.47–94.96	82.86–94.20	All significant at 1% or 5% probability levels
MG VIII	MG VII, MG VIII, and MG IX	90.03–95.08	80.71–94.51	All significant at 1% or 5% probability levels except for sample size 1000

### 3.3 The plateau of captured diversity in DS

For the *G. max* entire collection and the *G. soja* entire collection, the captured diversity plateaued when sample size was approximately 500 and 700 in DS, respectively (Figure 1). For MGs MG 00, 0, II, III, VI, VII, and VIII, diversity plateaus were reached when sample size was approximately 300. For MG IV and MG IX, diversity plateaus were reached when sample size was approximately 400. For MG V, a diversity plateau was reached when sample size was approximately 200 (Table S2).

### 3.4 Examples of a DS to eliminate highly similar accessions and to retain genetic diversity of traits, maturity groups, and geographical origin

We used Table S1 to randomly select a DS of 1849 accessions for *G. max*. After testing the genetic distances among accessions, no pair of accessions had a distance of 0 (identical), and only 109 pairs of accessions had a distance less than 0.10. For RS, 594 pairs of accessions had a distance of 0, and a total of 23,785 pairs of accessions had a distance less than 0.10. For the *G. soja* DS with 116 accessions, there were 0 pairs with 0 distance, and only one pair with a distance less than 0.10. However, for the *G. soja* RS, there were 17 pairs with 0 distance, and 564 pairs with a distance less than 0.10. The average distance was higher in DS than in RS for both *G. max and G. soja* (Table 4).

**TABLE 4:** Average frequencies of pair‐wise genetic distances among 10 sets of 1849 G. max diverse accessions and random accessions, and 10 sets of 116 *G. soja* diverse accessions and random accessions.

Pair‐wise distance range	Ten sets of 1849 *G. max* diverse sets	Ten sets of 1849 *G. max* random sets	Ten sets of 116 *G. soja* diverse sets	Ten sets of 116 *G. soja* random sets
0	0	594	0	17
0–0.05	9	13,226	0	416
0.05–0.10	100	9965	1	132
0.10–0.15	1443	49,290	8	250
0.15–0.20	16,369	398,169	30	548
0.20–0.25	231,220	1,585,132	1634	5175
0.25–0.30	2,419,333	3,959,651	35,685	33,920
0.30–0.35	8,093,155	6,446,288	19,813	21,382
0.35–0.40	5,484,903	3,752,090	7206	4519
0.40–0.45	741,292	701,302	1630	261
0.45–0.50	96,936	169,053	693	80
Mean	0.337	0.316	0.305	0.292
Standard deviation	0.039	0.056	0.041	0.045

*G. max* DS included accessions from all countries and MGs (Table S3). The means and standard deviations for quantitative traits such as protein content, oil content, and seed yield in the *G. max* DS were very similar to those in the entire *G. max* collection. The proportions of accessions differing in pest and disease resistance (Table S4), stem termination type, flower color, seed coat color, pubescence color, hilum color (Table S5), and flowering date and maturity date (Table S6) were also very similar between the *G. max* DS and the entire *G. max* collection. Similar results were also observed between the *G. soja* DS and the entire G. s*oja* collection (Table S7‐S12). Although trait data for accessions were collected across different locations and/or years, the comparison of retained genetic diversity between the entire collection and the DS of 1849 accessions remains valid, given that the DS mimicked the MGs and geographic origins of the entire collection.

### 3.5 Effectiveness of DS at capturing genetic diversity from SNPs in the 1511 resequenced soybean genomes

A subset of 42,509 random SNPs was selected from a dataset of 1511 whole‐genome sequences. These SNPs were used to develop 10 cultivated soybean DS with sample sizes of 10, 25, 50, and 100, respectively. These SNPs were found to retain an average of 77%, 90%, 96%, and 99% of the genetic diversity of 1308 cultivated soybean accessions, respectively, and an average of 57%, 78%, 90%, and 98% of the genetic diversity of 203 wild soybeans, respectively. Based on the full set of 6,230,008 SNPs from the 1308 resequenced cultivated soybean lines, the same 10 DS with sample sizes of 10, 25, 50, and 100 soybean accessions retained 77%, 90%, 96%, and 99% of the genetic diversity. Meanwhile, based on the full set of 13,311,173 SNPs found among 203 wild soybeans, 10 DS with sample sizes of 10, 25, 50, and 100 accessions could retain 57%, 78%, 90%, and 98% of the genetic diversity, which was surprisingly consistent with the prediction results based on the subset of 42,509 SNPs (Tables 5 and 6). Likewise, DS captured significantly higher diversity than RS for both wild and cultivated soybeans in almost all cases, especially at smaller sample sizes (Tables 5 and 6).

**TABLE 5:** Average number of single‐nucleotide polymorphisms (SNPs) and diversity retained in *G. max* and *G. soja* for 10 random sets and 10 diverse sets based on a 42,509 SNP subset of the 1.5K soybean genomes.

Sample size	Average number of SNPs in 10 random *G. soja* sets based on 42,509 SNPs	Diversity retained	Average number of SNPs in 10 random *G. max* sets based on 42,509 SNPs	Diversity retained	Average number of SNPs in 10 *G. soja* diverse sets based on 42,509 SNPs	Diversity retained	Average number of SNPs in 10 *G. max* diverse sets based on 42,509 SNPs	Diversity retained
*k* = 10	21,417 ± 1130.7	0.5	26,534 ± 1472.8	0.62	24,063 ± 665.4	0.57	32,768 ± 347.0	0.77
*k* = 25	31,137 ± 996.7	0.73	33,102 ± 831.9	0.78	33,047 ± 248.4	0.78	38,167 ± 240.0	0.9
*k* = 50	37,479 ± 756.0	0.88	38,168 ± 526.1	0.90	38,235 ± 339.7	0.90	40,692 ± 116.7	0.96
*k* = 100	41,546 ± 143.8	0.98	40,445 ± 481.7	0.95	41,636 ± 190.9	0.98	41,888 ± 65.9	0.99

**TABLE 6:** Average number of single‐nucleotide polymorphisms (SNPs) and diversity retained in *G. max* and *G. soja* for 10 random sets and 10 diverse sets based on all SNPs of the 1.5K soybean genome sequence.

Sample size	Average number of SNPs in 10 random *G. soja* sets based on 13,311,173 SNPs from 203 *G. soja* genomes	Diversity retained	Average number of SNPs in 10 random *G. max* sets based on 6,230,008 SNPs from 1308 *G. max* genomes	Diversity retained	Average number of SNPs in 10 diverse *G. soja* sets based on 13,311,173 SNPs from 203 *G. soja* genomes	Diversity retained	Average number of SNPs in 10 diverse *G. max* sets based on 6,230,008 SNPs from 1308 *G. max* genomes	Diversity retained
*k* = 10	6,717,607 ± 348,233.4	0.5	3,891,137 ± 225,864.1	0.62	7,527,210 ± 233,678.4	0.57	4,805,637 ± 52,073.5	0.77
*k* = 25	9,760,226 ± 300,912.4	0.73	4,859,538 ± 127,963.5	0.78	10,365,972 ± 80,991.4	0.78	5,604,082 ± 37,446.5	0.9
*k* = 50	11,737,549 ± 242,654.3	0.88	5,597,610 ± 84,566.7	0.9	11,980,723 ± 112,327.9	0.9	5,970,224 ± 15,718.8	0.96
*k* = 100	13,011,245 ± 45,883.6	0.98	5,930,670 ± 77,352.1	0.95	13,034,547 ± 68,948.4	0.98	6,141,361 ± 8936.1	0.99

### 3.6 Assessing genetic diversity in soybean core accessions selected based on phenotypic traits and passport information

Among the core collection selected by Oliveira et al. (2010), 72 pairs exhibited zero genetic distance (i.e., 100% similarity), while 614 pairs had genetic distances of less than 5% (Table 7), involving a total of 488 accessions. Selection based solely on phenotypic traits cannot effectively eliminate genetically identical or highly similar accessions due to the limited number of observable phenotypic characteristics.

**TABLE 7:** Pair‐wise genetic distance among 1685 core accessions previously reported based on the analysis of markers in the SoySNP50K assay.

Genetic distance range	Number of accession pairs
0	72
0–0.05	614
0.05–0.10	1076
0.10–0.15	3367
0.15–0.20	27,836
0.20–0.25	106,040
0.25–0.30	299,317
0.30–0.35	436,693
0.35–0.40	311,511
0.40–0.45	138,235
0.45–0.50	48,892
Mean	0.33

### 3.7 Instruction for using Soy‐DS Selector

The DS selections described in the sections above were performed with our Soy‐DS Selector tool. This tool can be used to help researchers create optimized DS sets from the USDAARS Soybean Germplasm Collection, including both wild and cultivated accessions. The tool allows selection by MG, sample size, seed availability, and user preferences, ensuring maximum genetic diversity with minimal redundancy. The Soy‐DS Selector is provided in Table S1. To use it, follow these steps. *Step 1*: Choose the column that matches the desired size and germplasm type of your DS. Note that the entire *G. max* collection is in spreadsheet columns G‐S, *G. max* within different MGs is in columns T‐FD, and the entire *G. soja* collection is in columns FE‐FQ. *Step 2*: If selecting DS by MG, be sure to identify columns for MG (e.g., MG 0, MG I, MG II) and the desired number of accessions. *Step 3*: Sort the table by clicking on the dropdown arrow of the chosen column header and selecting the “Sort largest to smallest option.” The genotypes will sort according to clusters of similarity (e.g., genotypes from cluster “25” are all similar to one another). *Step 4*: Select one accession per cluster (e.g., one from cluster “0”, one from cluster “1”) until one accession from each cluster is represented; the selected genotypes compose your DS. If seeds of the selected accession are not available or fail to germinate, another accession from the same cluster can be selected as a replacement. Users can also select an accession they prefer from each cluster. The selected set is expected to exclude genetically similar germplasm and maximize genetic diversity.

## 4 DISCUSSION

Most studies indicate that the soybean originated in China (Dupare et al., 2008; Hymowitz, 2008) and has spread around the world. The USDA Soybean Germplasm Collection includes germplasm from 87 countries (Song et al., 2015). Since this germplasm cannot be differentiated by morphological or agronomic traits, it is expected that some of them are identical or highly similar in nature. Creation of diverse soybean germplasm from the collection can reduce the number of germplasm resources that researchers need to study, cultivate, and maintain, thereby improving resource utilization efficiency. By focusing on manageable, diverse germplasm subsets, scientists can focus on the detailed characterization, evaluation, and utilization of genetic traits relevant to their breeding programs. Diverse germplasm sets serve as a representative sample that preserves the genetic diversity of the entire germplasm collection as much as possible. They ensure that valuable traits such as disease resistance, stress tolerance, and nutritional quality are preserved for breeding efforts and genetic research. Diverse germplasm sets can be used for a variety of purposes, including phenotypic evaluation and genomic studies, identification of markers or loci associated with important traits or genes controlling the traits, and development of breeding populations aimed at improving soybean productivity and its ability to cope with biotic and abiotic stresses. Using the SoySNP50K dataset, we calculated the genetic distances among all 1685 core accessions selected by Oliveira et al. (2010) based on phenotypic traits, and found that redundant or genetically highly similar accessions were included in the core collection. The results showed that phenotypic traits alone may not be sufficient to construct a good core set. In contrast, using SNP markers that are evenly distributed across the genome may better reflect genetic diversity and eliminate redundant or highly similar accessions (Table 4), compared to relying on a limited number of soybean phenotypic traits and geographic origin information. We should also note that although the 50K SNP panel can distinguish most highly similar accessions, some redundant accessions may still differ phenotypically, for example, within this germplasm collection, some near‐isogenic lines exist. Among the 291 near‐isogenic line “IClark” accessions, 36 showed a similarity higher than 99.9%, and four showed 100% similarity; among the 139 “IHarosoy” germplasm accessions, 37 showed a similarity higher than 99.9%, and two showed 100% similarity; among the 100 “IWilliams” germplasm accessions, 29 showed a similarity higher than 99.9%, and three showed 100% similarity. Although the 50K SNP panel can distinguish most highly similar accessions, some redundant accessions may still differ phenotypically. Furthermore, natural cross‐pollination and variation within germplasm resources may lead to some heterozygosity within the germplasm (Jiang et al., 2024), and there is also known heterogeneity within some accessions (Mihelich et al., 2020). Some of the genetically highly similar germplasm resources may still have value.

Several strategies have been proposed for the selection of core sets after stratification. These include the marker‐based H strategy (after Nei's index of gene diversity), the M strategy (marker allele richness) (Schoen & Brown, 1993), the non‐marker‐based C strategy (sampling of a constant number of accessions per region), and other strategies (Chandra et al., 2002; Jansen & Van Hintum, 2007; Zeuli & Qualset, 1993). Schoen and Brown (1993) compared the maximal number of alleles (allelic richness) retained in collections of different species among some strategies and concluded that marker‐assisted strategies yielded higher overall allelic richness in the simulated collections than other strategies and were particularly effective in conserving geographically localized alleles. The marker‐assisted approach is especially appropriate in inbred species. Although the stratified sampling strategy is proven efficient, non‐stratified procedures have also been used to select core sets from entire collections (De Beukelaer et al., 2012; Jansen & Van Hintum, 2007; Wang et al., 2007). Several software programs have been used for core sample selection, such as MSTRAT (Gouesnard et al., 2001), PowerCore (Kim et al., 2007), and Core Hunter 3 (Thachuk et al., 2009). MSTRAT was developed based on the M strategy. This approach examines all possible core collections and finds the core set maximizing the number of observed alleles at the marker loci. PowerCore is also based on the M strategy; however, instead of examining all possible combinations, it uses a heuristic algorithm to solve the traveling salesman problem. Both MSTRAT and PowerCore can produce very similar results (Jeong et al., 2017). The Core Hunter strategy, based on stochastic local search methods (Replica Exchange Monte Carlo and Mixed Replica search methods), was developed to address problems of multiple genetic measures and a large number of possible core subsets. However, current methods and procedures are either unable to analyze such large datasets or are not flexible enough to meet researchers' needs and applications, such as accession preference, substitution, and ease of use. We performed simulations using the SoySNP50K dataset and a whole‐genome sequence SNP dataset, our results show that the DS selection method, which uses PLINK to cluster accessions and select one accession from each cluster, is highly effective in preserving accession genetic diversity. This method not only outperforms random selection but also accurately predicts the genome‐wide genetic diversity that DS can capture. Because soybean germplasm is photoperiod‐sensitive and can only flower and mature normally within a narrow range of MGs, we also developed DS for each MG, which will help researchers conduct studies across different MGs. In our initial analysis, we calculated the average genetic distance of each accession to all other accessions and selected the accession with the largest average genetic distance from each cluster to construct the optimal diverseity set. However, this approach did not result in a significant improvement in capturing genetic diversity compared to randomly selected sets. We also observed no significant differences in genetic diversity among different DS generated through permutation. Therefore, users can select any germplasm from each cluster based on their specific criteria without substantially affecting the overall genetic diversity of the diverseity set, as germplasm within each cluster is genetically highly similar, and the overall genetic diversity of soybean or wild soybean is generally low. If a DS sample size beyond the ones listed in Table S1 is required, users can also develop their own DS using this method, as the SoySNP50K dataset and PLINK software are publicly available.

Using the Soy‐DS Selector tables created or the methods described in this study, we have selected multiple diverse sets for soybean researchers across different soybean‐growing regions in the United States. These diverse collections have been used for resequencing (Valliyodan et al., 2021), GWAS of traits such as seed protein content, oil content, amino acid and fatty acid content (J. Zhang et al., 2018), seed sugar composition (Hu et al., 2023), as well as for identifying novel loci and candidate genes for resistance to frogeye leaf spot (McDonald et al., 2023) and white mold (Wen et al., 2018), seed weight (J. Zhang et al., 2016), and other agronomic traits (J. Zhang et al., 2015). The diverse accessions have also been used to study the genetic diversity of seed composition traits in wild soybean (La et al., 2019), the evolutionary analysis of the *GmNNL1* gene that restricts symbiotic compatibility (B. Zhang et al., 2021), nodule occupancy by different rhizobia strains (Araya et al., 2023), the identification of seed traits associated with high test weight (Shea et al., 2023), the evaluation of potential for genomic selection for methionine content in soybean seeds (Singer et al., 2022), and the development of recombinant inbred populations (Chen et al., 2025). The information provided by this study is expected to facilitate the utilization of germplasm resources by offering a user‐customizable table that allows researchers to select diverse soybean germplasm accessions from the USDA Soybean Germplasm Collection. This tool enables users to filter out genetically similar accessions, making it highly practical and efficient for targeted research. For example, a researcher conducting a GWAS on protein content can select germplasm representing the broadest genetic variation, including wild relatives, landraces, and known high‐ and low‐protein lines. Similarly, a breeder aiming to identify drought‐tolerant or disease‐resistant germplasm can select accessions that capture the full diversity of the collection—something difficult to achieve otherwise—spanning multiple genetic backgrounds, or construct a diverse germplasm set for genomic prediction. By providing this flexibility, the table transforms germplasm utilization from a static resource into a dynamic, goal‐driven process. Wild soybean harbors valuable genes related to production performance that are absent from the cultivated soybean gene pool. Developing DS of wild soybean will greatly facilitate the utilization of these genes in breeding programs. Furthermore, incorporating diverse sets of both cultivated and wild soybeans is essential for maximizing genetic variation, uncovering novel alleles, and leveraging complementary traits—such as high yield from cultivated types and stress tolerance from wild relatives—to enhance productivity, resilience, and adaptability in soybean improvement.

## AUTHOR CONTRIBUTIONS

**Qijian Song**: Conceptualization; formal analysis; funding acquisition; investigation; methodology; writing—original draft; writing—review and editing. **Susan Araya**: Formal analysis; writing—review and editing. **Chuck Quigley**: Resources; writing—review and editing. **Patrick Elia**: Writing—review and editing.

## CONFLICT OF INTEREST STATEMENT

The authors declare no conflicts of interest.

## Supporting information




**Table S1**. Soy‐DS Selector cluster table used to build user‐selectable diverse collections within maturity groupos, based on the entire *Glycine max* and *Glycine soja* collections.

**Table S2**. Average number of SNPs and the percentage of diverstiy ratained from 10 diverse sets and 10 random sets, as well as the test of difference in the percentage of diversity retained between the diverse set and random set

**Table S3** Comparison of the USDA *Glycine max* germplasm collection and a diverse set of 1,849 accessions in terms of the number of accessions from different geographic origins and maturity groups

**Table S4** Comparison of the USDA *Glycine max* germplasm collection and a diverse set of 1,849 accessions in terms of the percentage of accessions for pest and disease resistance

**Table S5** Comparison of the USDA *Glycine max* germplasm collection and a diverse set of 1,849 accessions in terms of the percentage of accessions for growth habit and morphological characteristics

**Table S6** Comparison of the USDA *Glycine max* germplasm collection and a diverse set of 1,849 accessions in terms of the proportion of accessions at different flowering and maturity dates

**Table S7** Comparison of the USDA *Glycine max* germplasm collection and a diverse set of 1,849 accessions in terms of quantitative traits

**Table S8** Comparison of the USDA *Glycine soja* germplasm collection and a diverse set of 116 accessions in terms of the number of accessions from different geographic origins and maturity groups

**Table S9** Comparison of the USDA *Glycine soja* germplasm collection and a diverse set of 116 accessions in terms of the percentage of accessions for pest and disease resistance

**Table S10** Comparison of the USDA *Glycine soja* germplasm collection and a diverse set of 116 accessions in terms of the percentage of accessions for growth habit and morphological characteristics

**Table S11** Comparison of the USDA *Glycine soja* germplasm collection and a diverse set of 116 accessions in terms of the proportion of accessions at different flowering and maturity dates

**Table S12** Comparison of the USDA *Glycine soja* germplasm collection and a diverse set of 116 accessions in terms of quantitative traits

## Data Availability

The SoySNP50K SNP dataset for soybean germplasm resources is available at SoyBase (funded by the USDA Agricultural Research Service) at https://www.soybase.org/tools/snp50k/. All other datasets are listed in the Supporting Information to this paper.
