# Supplementary material for: Development of user‐selectable diverse sets of cultivated and wild soybean germplasm for genetic and breeding applications
Source: Plant Genome. 2026 Mar 9;19(1):e70216. doi: 10.1002/tpg2.70216 (PMC12968749; doi:10.1002/tpg2.70216)
Supplement: Supplementary file 3 — Table S3 Comparison of the USDA Glycine max germplasm collection and a diverse set of 1,849 accessions in terms of the number of accessions from different geographic origins and maturity groups [file TPG2-19-e70216-s003.docx]

**Table S3** Comparison of the USDA *Glycine max* germplasm collection and a diverse set of 1,849 accessions in terms of the number of accessions from different geographic origins and maturity groups

| ***Geographic origin*** | ***Number of accessions in G. max* collection** | ***Number of accessions in G. max* diverse set** |
| --- | --- | --- |
| China | 6,185 | 1,009 |
| Korea | 3,614 | 127 |
| Japan | 2,934 | 127 |
| Russia | 637 | 83 |
| United States | 1,582 | 188 |
| Others | 3,381 | 291 |
| Unknown | 151 | 24 |
| Total | 18,484 | 1,849 |

| ***Maturity group*** | ***Number of accessions in G. max* collection** | ***Number of accessions in G. max* diverse set** |
| --- | --- | --- |
| 000 | 141 | 10 |
| 00 | 513 | 43 |
| 0 | 1,116 | 90 |
| I | 1,673 | 187 |
| II | 2,061 | 211 |
| III | 1,974 | 275 |
| IV | 4,232 | 402 |
| V | 2,517 | 187 |
| VI | 1,512 | 139 |
| VII | 928 | 117 |
| VIII | 947 | 106 |
| IX | 726 | 63 |
| X | 106 | 11 |
| Unknown | 37 | 8 |
| Total | 18,484 | 1,849 |
