# Supplementary material for: Development of user‐selectable diverse sets of cultivated and wild soybean germplasm for genetic and breeding applications
Source: Plant Genome. 2026 Mar 9;19(1):e70216. doi: 10.1002/tpg2.70216 (PMC12968749; doi:10.1002/tpg2.70216)
Supplement: Supplementary file 4 — Table S4 Comparison of the USDA Glycine max germplasm collection and a diverse set of 1,849 accessions in terms of the percentage of accessions for pest and disease resistance [file TPG2-19-e70216-s001.docx]

**Table S4** Comparison of the USDA *Glycine max* germplasm collection and a diverse set of 1,849 accessions in terms of the percentage of accessions for pest and disease resistance

| ***Pests and diseases resistance*** | ***Percentage of accessions in G. max* collection** | ***Percentage of accessions in G. max* diverse set** |
| --- | --- | --- |
| **Soybean Aphid Resistance (aphid)** |  |  |
| Mostly Resistant | 0.7% | 1.1% |
| Resistant | 0.4% | 0.3% |
| Susceptible | 98.9% | 98.7% |
| **Cyst Nematode (nematcyst)** |  |  |
| Moderately Resistant | 2.2% | 4.2% |
| Moderately Susceptible | 27.8% | 32.0% |
| Resistant | 0.5% | 0.9% |
| Susceptible | 69.5% | 63.0% |
| **Soybean Sudden Death Syndrome (SDS)** |  |  |
| Mostly Resistant | 0.3% | 0.6% |
| Mostly Susceptible | 1.3% | 1.0% |
| Susceptible | 98.5% | 98.3% |
| **Phytophthora Rot (phytorot)** |  |  |
| Heterogeneous | 13.2% | 16.6% |
| Resistant | 36.6% | 28.9% |
| Susceptible | 50.3% | 54.5% |
