# Supplementary material for: Development of user‐selectable diverse sets of cultivated and wild soybean germplasm for genetic and breeding applications
Source: Plant Genome. 2026 Mar 9;19(1):e70216. doi: 10.1002/tpg2.70216 (PMC12968749; doi:10.1002/tpg2.70216)
Supplement: Supplementary file 5 — Table S5 Comparison of the USDA Glycine max germplasm collection and a diverse set of 1,849 accessions in terms of the percentage of accessions for growth habit and morphological characteristics [file TPG2-19-e70216-s012.docx]

**Table S5** Comparison of the USDA *Glycine max* germplasm collection and a diverse set of 1,849 accessions in terms of the percentage of accessions for growth habit and morphological characteristics

| ***Growth habit and morphological characteristics*** | ***Percentage of accessions in G. max* collection** | ***Percentage of accessions in G. max* diverse set** |
| --- | --- | --- |
| **Stem termination type (stemterm)** |  |  |
| Determinate | 50% | 31% |
| Indeterminate | 42% | 61% |
| Semi-determinate | 8% | 8% |
| **Flower color (flwcolor)** |  |  |
| Purple | 65.6% | 58.7% |
| White | 33.5% | 40.7% |
| Others | 0.9% | 0.7% |
| **Seed coat color (scoatcolor)** |  |  |
| Black | 10% | 9% |
| Brown | 4% | 5% |
| Green | 9% | 8% |
| Yellow | 72% | 73% |
| Others | 5% | 6% |
| **Pubescence color (pubcolor)** |  |  |
| Gray | 45% | 42% |
| Tawny | 50% | 52% |
| Others | 5% | 7% |
| **Hilum color (hilumcolor)** |  |  |
| Buff | 20% | 24% |
| Black | 20% | 23% |
| Brown | 22% | 23% |
| Yellow | 20% | 11% |
| Others | 18% | 19% |
