# Supplementary material for: Development of user‐selectable diverse sets of cultivated and wild soybean germplasm for genetic and breeding applications
Source: Plant Genome. 2026 Mar 9;19(1):e70216. doi: 10.1002/tpg2.70216 (PMC12968749; doi:10.1002/tpg2.70216)
Supplement: Supplementary file 6 — Table S6 Comparison of the USDA Glycine max germplasm collection and a diverse set of 1,849 accessions in terms of the proportion of accessions at different flowering and maturity dates [file TPG2-19-e70216-s007.docx]

**Table S6** Comparison of the USDA *Glycine max* germplasm collection and a diverse set of 1,849 accessions in terms of the proportion of accessions at different flowering and maturity dates

| **Flowering date** | ***Percentage of accessions in G. max* collection** | ***Percentage of accessions in G. max* diverse set** | **Maturity date** | ***Percentage of accessions in G. max* collection** | ***Percentage of accessions in G. max* diverse set** |
| --- | --- | --- | --- | --- | --- |
| 6/09 - 6/30 | 14.8% | 9.4% | 7/31 - 8/31 | 2.6% | 2.1% |
| 7/01 - 7/31 | 66.1% | 71.1% | 9/01 - 9/30 | 53.0% | 51.4% |
| 8/01 - 8/31 | 18.5% | 19.1% | 10/01 - 10/31 | 40.6% | 43.0% |
| 9/01 - 9/30 | 0.6% | 0.4% | 11/01 - 12/14 | 3.8% | 3.5% |
