# Supplementary material for: Development of user‐selectable diverse sets of cultivated and wild soybean germplasm for genetic and breeding applications
Source: Plant Genome. 2026 Mar 9;19(1):e70216. doi: 10.1002/tpg2.70216 (PMC12968749; doi:10.1002/tpg2.70216)
Supplement: Supplementary file 7 — Table S7 Comparison of the USDA Glycine max germplasm collection and a diverse set of 1,849 accessions in terms of quantitative traits [file TPG2-19-e70216-s011.docx]

**Table S7** Comparison of the USDA *Glycine max* germplasm collection and a diverse set of 1,849 accessions in terms of quantitative traits

|  | ***G. max* collection** | | | | ***G. max* diverse set** | | | |
| --- | --- | --- | --- | --- | --- | --- | --- | --- |
| ***Traits*** | **Accessions** | **Mean** | **Range** | **SD** | **Accessions** | **Mean** | **Range** | **SD** |
| Oil content | 16,125 | 17.9 | 7.6 ̶ 24.3 | 2.0 | 1,614 | 17.7 | 7.6 ̶ 23.5 | 2.2 |
| Protein content | 16,125 | 44.0 | 31.7 ̶ 57.9 | 2.7 | 1,614 | 44.0 | 33.1 ̶ 54.2 | 2.9 |
| Height | 15,433 | 87.2 | 20 ̶ 999 | 36.4 | 1,523 | 100.8 | 20 ̶ 999 | 37.3 |
| Yield | 15,358 | 2.0 | 0.02 ̶ 4.48 | 0.7 | 1,521 | 2.0 | 0.08 ̶ 4.48 | 0.8 |
