# Supplementary material for: Development of user‐selectable diverse sets of cultivated and wild soybean germplasm for genetic and breeding applications
Source: Plant Genome. 2026 Mar 9;19(1):e70216. doi: 10.1002/tpg2.70216 (PMC12968749; doi:10.1002/tpg2.70216)
Supplement: Supplementary file 8 — Table S8 Comparison of the USDA Glycine soja germplasm collection and a diverse set of 116 accessions in terms of the number of accessions from different geographic origins and maturity groups [file TPG2-19-e70216-s005.docx]

**Table S8** Comparison of the USDA *Glycine soja* germplasm collection and a diverse set of 116 accessions in terms of the number of accessions from different geographic origins and maturity groups

| ***Geographic origin*** | ***Number of accessions in G. soja* collection** | ***Number of accessions in G. soja* diverse set** |
| --- | --- | --- |
| China | 191 | 16 |
| Korea | 345 | 55 |
| Japan | 294 | 31 |
| Russia | 270 | 7 |
| Others | 68 | 7 |
| Total | 1,168 | 116 |

| ***Maturity group*** | ***Number of accessions in G. soja* collection** | ***Number of accessions in G. soja* diverse set** |
| --- | --- | --- |
| 000 | 108 | 2 |
| 00 | 49 | 1 |
| 0 | 52 | 0 |
| I | 60 | 4 |
| II | 95 | 8 |
| III | 50 | 4 |
| IV | 84 | 8 |
| V | 356 | 51 |
| VI | 166 | 25 |
| VII | 79 | 7 |
| VIII | 1 | 0 |
| IX | 3 | 0 |
| X | 4 | 0 |
| Unknown | 61 | 6 |
| Total | 1,168 | 116 |
