# Supplementary material for: Development of user‐selectable diverse sets of cultivated and wild soybean germplasm for genetic and breeding applications
Source: Plant Genome. 2026 Mar 9;19(1):e70216. doi: 10.1002/tpg2.70216 (PMC12968749; doi:10.1002/tpg2.70216)
Supplement: Supplementary file 9 — Table S9 Comparison of the USDA Glycine soja germplasm collection and a diverse set of 116 accessions in terms of the percentage of accessions for pest and disease resistance [file TPG2-19-e70216-s006.docx]

**Table S9** Comparison of the USDA *Glycine soja* germplasm collection and a diverse set of 116 accessions in terms of the percentage of accessions for pest and disease resistance

| ***Pests and diseases resistance*** | ***Percentage of accessions in G. soja* collection** | ***Percentage of accessions in G. soja* diverse set** |
| --- | --- | --- |
| **Cyst Nematode (nematcyst)** |  |  |
| Moderately Resistant | 0.7% | 3.0% |
| Moderately Susceptible | 51.1% | 53.5% |
| Resistant | 0.3% | 0.0% |
| Susceptible | 47.9% | 43.4% |
| **Phytophthora Rot (phytorot)** |  |  |
| Resistant | 9.4% | 12.5% |
| Susceptible | 90.6% | 87.5% |
