# Supplementary material for: Development of user‐selectable diverse sets of cultivated and wild soybean germplasm for genetic and breeding applications
Source: Plant Genome. 2026 Mar 9;19(1):e70216. doi: 10.1002/tpg2.70216 (PMC12968749; doi:10.1002/tpg2.70216)
Supplement: Supplementary file 10 — Table S10 Comparison of the USDA Glycine soja germplasm collection and a diverse set of 116 accessions in terms of the percentage of accessions for growth habit and morphological characteristics [file TPG2-19-e70216-s009.docx]

**Table S10** Comparison of the USDA *Glycine soja* germplasm collection and a diverse set of 116 accessions in terms of the percentage of accessions for growth habit and morphological characteristics

| ***Growth habit and morphological characteristics*** | ***Percentage of accessions in G. soja* collection** | ***Percentage of accessions in G. soja* diverse set** |
| --- | --- | --- |
| **Stem termination type (stemterm)** |  |  |
| Determinate | 20% | 23% |
| Indeterminate | 80% | 77% |
| **Flower color (flwcolor)** |  |  |
| Purple | 99.8% | 100% |
| Dark purple | 0.2% | 0.0% |
| **Leaflet shape of *Glycine soja* (leafshape)** |  |  |
| Lanceolate | 8.3% | 1.0% |
| Linear | 5.1% | 2.1% |
| Oval | 62.5% | 70.1% |
| Ovate | 22.9% | 26.8% |
| Ultralinear | 1.2% | 0.0% |
| **Seed coat luster (scoatlust)** |  |  |
| Bloom | 68.4% | 52.5% |
| Dull | 16.1% | 32.5% |
| Dense bloom | 11.2% | 5.0% |
| Light bloom | 3.8% | 7.5% |
| Others | 0.6% | 2.5% |
