# Supplementary material for: Development of user‐selectable diverse sets of cultivated and wild soybean germplasm for genetic and breeding applications
Source: Plant Genome. 2026 Mar 9;19(1):e70216. doi: 10.1002/tpg2.70216 (PMC12968749; doi:10.1002/tpg2.70216)
Supplement: Supplementary file 11 — Table S11 Comparison of the USDA Glycine soja germplasm collection and a diverse set of 116 accessions in terms of the proportion of accessions at different flowering and maturity dates [file TPG2-19-e70216-s010.docx]

**Table S11** Comparison of the USDA *Glycine soja* germplasm collection and a diverse set of 116 accessions in terms of the proportion of accessions at different flowering and maturity dates

| **Flowering dates** | ***Percentage of accessions in G. soja* collection** | ***Percentage of accessions in G. soja* diverse set** | **Maturity dates** | ***Percentage of accessions in G. soja* collection** | ***Percentage of accessions in G. soja* diverse set** |
| --- | --- | --- | --- | --- | --- |
| 6/09 - 6/30 | 6.7% | 1.1% | 7/31 - 8/31 | 13.6% | 3.4% |
| 7/01 - 7/31 | 19.2% | 9.2% | 9/01 - 9/30 | 15.9% | 11.5% |
| 8/01 - 8/31 | 61.9% | 77.0% | 10/01 - 10/31 | 69.1% | 82.8% |
| 9/01 - 9/30 | 12.2% | 12.6% | 11/01 - 12/14 | 1.5% | 2.3% |
