# Supplementary material for: Development of user‐selectable diverse sets of cultivated and wild soybean germplasm for genetic and breeding applications
Source: Plant Genome. 2026 Mar 9;19(1):e70216. doi: 10.1002/tpg2.70216 (PMC12968749; doi:10.1002/tpg2.70216)
Supplement: Supplementary file 12 — Table S12 Comparison of the USDA Glycine soja germplasm collection and a diverse set of 116 accessions in terms of quantitative traits [file TPG2-19-e70216-s002.docx]

**Table S12** Comparison of the USDA *Glycine soja* germplasm collection and a diverse set of 116 accessions in terms of quantitative traits

|  | ***G. max* collection** | | | | ***G. max* diverse set** | | | |
| --- | --- | --- | --- | --- | --- | --- | --- | --- |
| ***Traits*** | **Accessions** | **Mean** | **Range** | **SD** | **Accessions** | **Mean** | **Range** | **SD** |
| Oil content | 993 | 11.00 | 7.5 - 16.5 | 1.23 | 99 | 11.1 | 8.5 - 16 | 1.4 |
| Protein content | 993 | 46.88 | 35.5 - 56.9 | 3.10 | 99 | 47.5 | 40.2 - 52.8 | 2.8 |
| Seed weight | 179 | 1.68 | 1 - 3.3 | 0.33 | 14 | 1.7 | 1.1 - 2.1 | 0.3 |
